# ­Morphological variations in the dorsal fin finlets of extant polypterids raise questions about their taxonomical validity

**DOI:** 10.7717/peerj.5083

**Published:** 2018-06-29

**Authors:** Marcos Vinícius Coelho, Camila Cupello, Paulo M. Brito

**Affiliations:** Departamento de Zoologia, Universidade do Estado do Rio de Janeiro, Rio de Janeiro, RJ, Brazil

**Keywords:** Pinnules, Polypteriforms, Morphological variation, Taxonomy

## Abstract

Fossil polypterids are mainly represented by disarticulated material, most of them pinnules. However, there is no study that proves the taxonomical validity of these structures. Here we describe the pinnules of four species of extant polypterids and report for the first time intraindividual variations in the pinnules according to their position in the dorsal fin. Nevertheless, when comparing two different specimens of one species there is little or no interindividual variation, suggesting that pinnule morphology may have taxonomical validity. As the fossil polypterid record is based mainly on the articular head of the pinnules, we suggest caution when describing new taxa, especially if different fragments corresponding to specific positions in the dorsal fin occur in the same locality.

## Introduction

The order Polypteriformes is known from the Triassic to the Recent and represents the sister group of all other extant actinopterygians (Patterson, 1982; Lauder & Liem, 1983; Gardiner, 1984; Friedman, 2015; Giles et al., 2017). Extant polypteriforms are represented by the unique African family, Polypteridae (Daget, 1949; Daget, 1950; Daget, 1958; Daget, 1986; Greenwood, 1984; Daget & Desoutter, 1983). The polypterids form a monophyletic assemblage of two nominal genera: *Polypterus* Lacépède, 1803, the bichirs, with about 13 species and the monospecific *Calamoichthys* Smith, 1866, the reed fish (= *Erpetoichthys*; for the taxonomic discussion see Rizzato & Bockmann, 2017).

Fossil polypterids are known from the Cretaceous, Tertiary and Quaternary of Africa (Stromer, 1936; Gayet & Meunier, 1996; Werner & Gayet, 1997; Dutheil, 1998; Dutheil, 1999; Otero et al., 2006) and the Cretaceous and Paleocene of South America (Gayet & Meunier, 1991; Gayet & Meunier, 1992; Gayet & Meunier, 1993; Meunier & Gayet, 1996; Meunier & Gayet, 1998; Daget et al., 2001; Dutra & Malabarba, 2001; Medeiros et al., 2014) and they are represented by 10 fossil nominal genera. Except for some rare exceptions (c.f., Dutheil, 1998; Dutheil, 1999; Otero et al., 2006), these fossils are disarticulated elements (e.g., scales, vertebrae and pinnules), and among them many are based on pinnule’s morphology (Gayet & Meunier, 1996; Werner & Gayet, 1997), since, in some few taxa, the pinnules had an asymmetrical morphology (Werner & Gayet, 1997).

However, there are no further studies that prove the viability of this character as genera or species level diagnosis. Here we describe the complete series of pinnules of four extant species of polypterids (c.f., *P. endlicherii*, Heckel, 1847; *P. palmas* Ayres, 1850; *C. calabaricus* Smith, 1866; and *P. delhezi* Boulenger, 1899), in order to test the viability of these anatomic structures as a tool for taxonomic diagnoses mainly in the study of fossils.

## Material and Methods

### Specimen information

The pinnules used in this study were removed from adult specimens of: *Polypterus delhezi* (UERJ-PNT 525 and UERJ-PNT 528), *Polypterus endlicherii* (UERJ-PNT 522), *Polypterus palmas* (UERJ-PNT 526), and *Calamoichthys calabaricus* (UERJ-PNT 527), all permanently housed in the collections of the Universidade do Estado do Rio de Janeiro and registered under the acronym UERJ-PNT.

### Cleaning procedure

The pinnules were cleaned mechanically by removing the soft tissue with a pointless needle.

### Scanning electron microscopy

For scanning electron microscopy, samples of *Polypterus delhezi* (UERJ-PNT 525 and 528), *P. endlicherii* (UERJ-PNT 522), *P. palmas* (UERJ-PNT 526), and *Calamoichthys calabaricus* (UERJ-PNT 527) were coated with carbon by the high vacuum vaporization process in the Desk-V metallizer (Denton Vacuum, Moorestown, NJ, USA). SEM images were obtained using JEOL JSM-6510LV SEM.

### Nomenclature

The anatomical nomenclature used in this study is based on Gayet, Meunier & Werner (1997).

## Results

**Figure 1:**
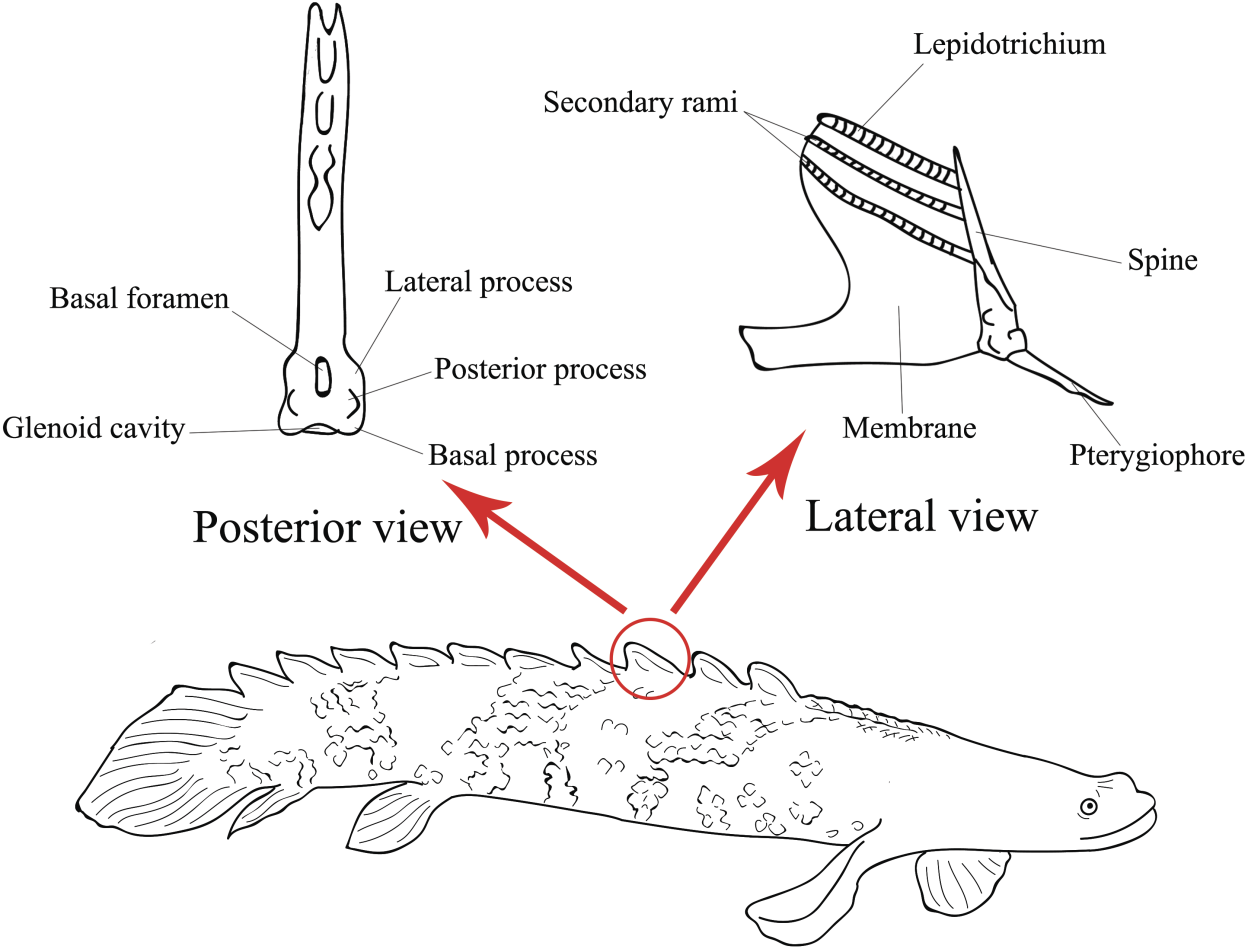
Illustration of the lateral view of a *Polypterus* specimen with anatomical details on lateral and posterior views of a pinnule (Modified from Gayet, Meunier & Werner, 1997).

The dorsal fin pinnules are somewhat symmetrical and composed of three parts (Gayet, Meunier & Werner, 1997): a spine that articulates with the pterygiophore, in its base are located the basal, lateral and posterior processes and the basal foramen, which is the exit point of the medular canal (Gayet, Meunier & Werner, 1997); a lepidotrichia fused in its basal part with the spine, where its distal part is free and divided into secondary rami; and a membrane that connects a finlet and its lepidotrichia to the anterior part of the next finlet (Fig. 1).

### P. delhezi

**Figure 2:**
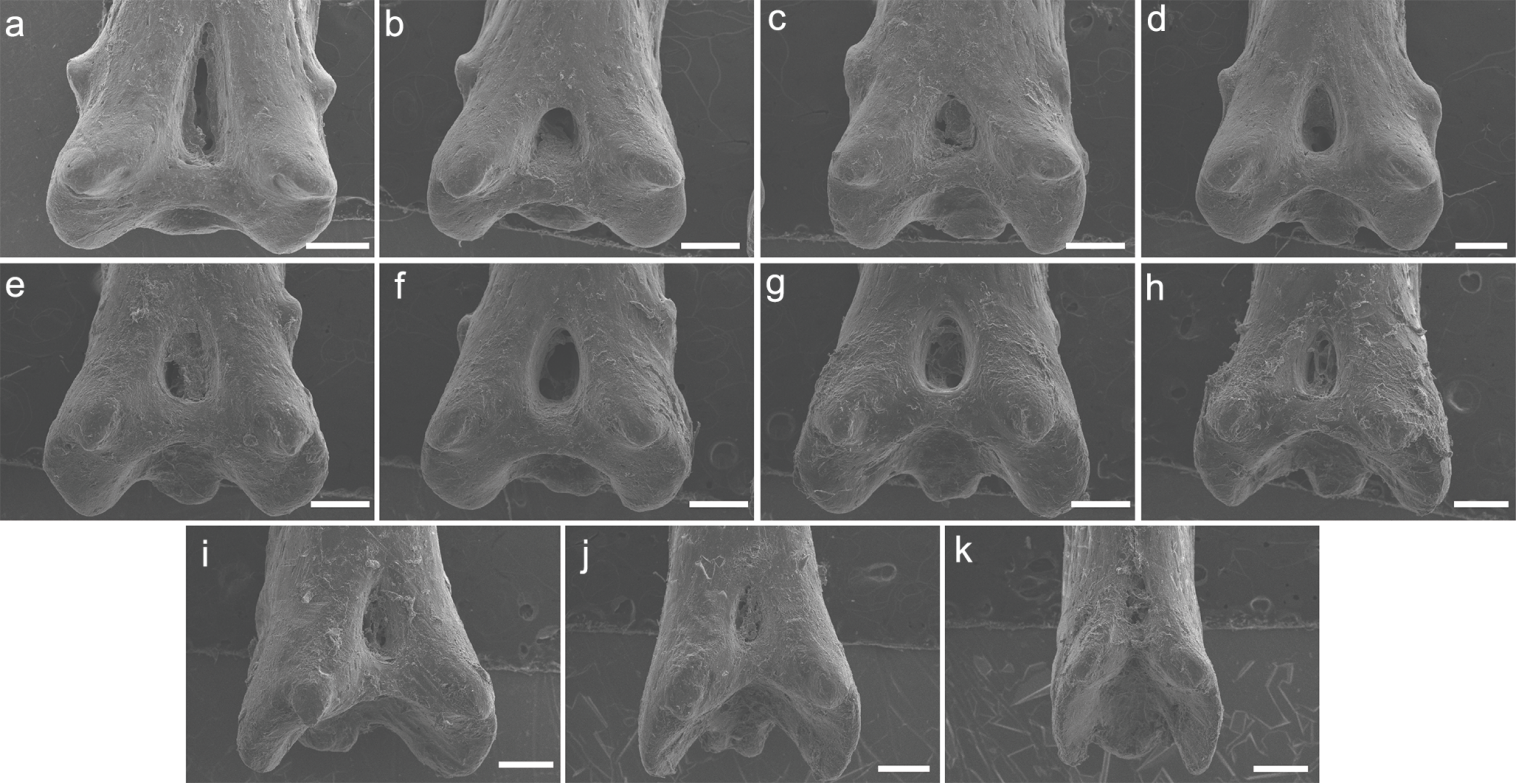
SEM images of the pinnulae of *P. delhezi.* UERJ-PNT 525. (A–K) corresponds to the 1st, 2nd, 3rd, 4th, 5th, 6th, 7th, 8th, 9th, 10th and 11th pinnulae, respectively. Scale = 500 µm).

Both specimens UERJ-PNT 525 and UERJ-PNT 528 had 11 pinnules and little or no difference was observed in the general morphology of the pinnules, remaining conservative according to its position in the dorsal fin (Fig. 2 and Fig. S1). The pinnules of *P. delhezi* present a well-defined basal process, a rounded basal foramen, a tiny posterior process, and a general trapezoid shape with a rounded and less-developed lateral process, when present. However, there are also some minor morphological differences among the different pinnules. Although almost all of them present a trapezoid shape (Figs. 2A–2J), formed by its spaced basal processes, the 11th pinnule displays a square shape (Fig. 2K). There is also a variation in the number of the lateral processes, while the 1st, 3rd, 4th and 6th pinnule (Figs. 2A, 2C, 2D, 2F) have slightly developed lateral processes in both sides, the 2nd, 5th and 7th pinnule (Figs. 2B, 2E, 2G) only display this process in one side, and the 8th, 9th, 10th and 11th pinnule do not present any lateral processes (pattern of the most posterior pinnules, Figs. 2H–2K). Also, in the 1st pinnule (Fig. 2A) the basal foramen is more elongated and higher than in the others, passing the upper limit of the lateral process. The others pinnules display a rounded and shorter basal foramen that does not pass the upper limit of the lateral process.

### P. endlicherii

**Figure 3:**
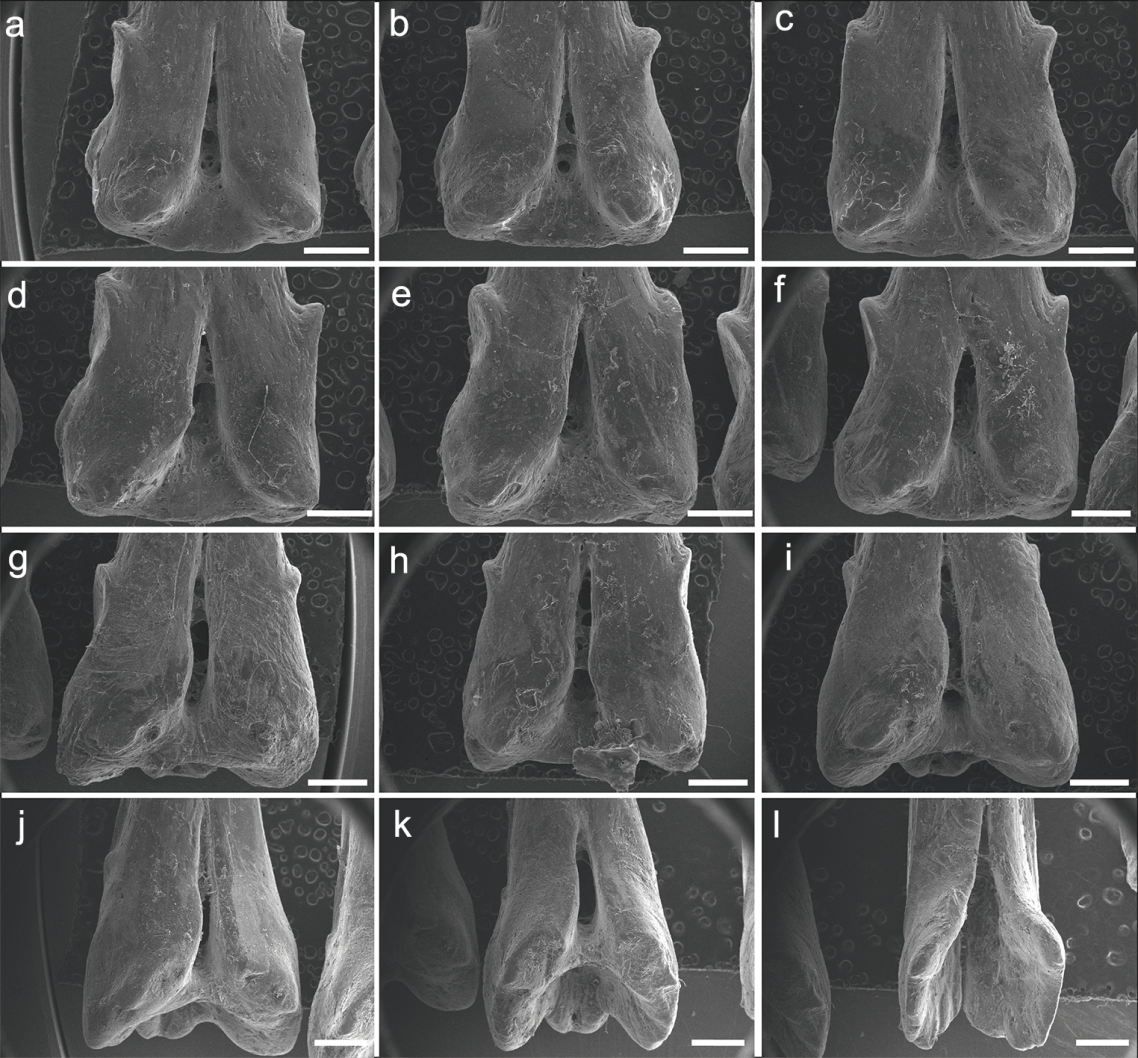
SEM images of the pinnules of *P. endlicherii* UERJ-PNT 522. (A–L) corresponds to the 1st, 2nd, 3rd, 4th, 5th, 6th, 7th, 8th, 9th, 10th, 11th and 12th pinnules, respectively. Scale = 1 mm).

The specimen UERJ-PNT 522 had 12 pinnules. The pinnules of *P. endlicherii* show great similarity among themselves (Fig. 3). The shape of the base is slightly trapezoid, keeping the same proportions; all basal processes are slightly rounded, except the 11th which displays a flattened process (Fig. 3K). The lateral processes have about the same size and shape (except for the 10th pinnule, which presents admittedly small lateral processes) and are present on both sides from the 1st to the 10th pinnules (Figs. 3A–3J), and absent in the 11th and 12th pinnules (Figs. 3K, 3L). The posterior processes are flattened and the 7th to the 11th pinnules present this process somewhat protuberant towards the foramen, at least in one side (Figs. 3G–3K); and the basal foramen is elongated. However, there are also some minor morphological differences, such as: the 1st and 6th pinnules present a rounded lower limit (Figs. 3A, 3F), while the 2nd, 3rd, 4th, 5th and 8th display a flat lower limit (Figs. 3B–3E, 3H), the 7th, 9th, 10th and 11th pinnules a concave limit (Figs. 3G, 3I–3K), and the 12th displays an open lower limit (there is no boundary between the basal foramen and the glenoid cavity, possibly a fusion between those structures) (Fig. 3L); the 1st, 2nd, 3rd and 7th display the upper limit of the basal foramen at the same height of the upper limit of the lateral processes (Figs. 3A–3C, 3G), while the 4th, 5th, 6th, 8th and 9th pinnules show the upper limit of the basal foramen below lateral processes (Figs. 3D–3F, 3H, 3I) and the 10th pinnule shows the upper limit of the basal foramen above lateral processes (Fig. 3J).

### P. palmas

**Figure 4:**
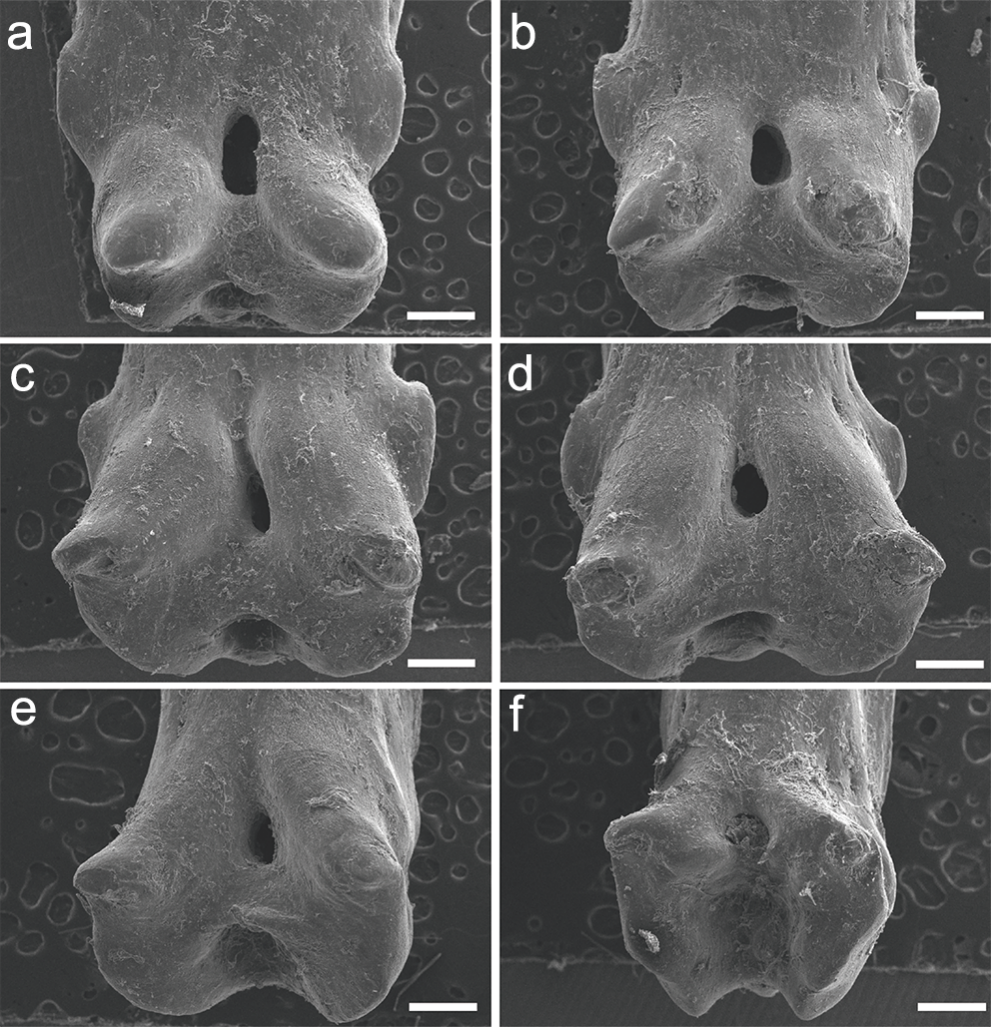
SEM images of the pinnules of *P. palmas.* UERJ-PNT 526. (A–F) corresponds to the 1st, 2nd, 4th, 5th, 6th and 7th pinnules, respectively. The 3rd pinnule was missing in this specimen. Scale = 500 µm).

The specimen UERJ-PNT 526 had seven pinnules, the third pinnule was missing. The pinnules do not display a uniform morphology, despite presenting some similarities, such as rounded basal foramen and protuberant basal processes with rounded edges (Fig. 4). Regarding the morphology of these pinnules, the following differences can be identified: the 1st and 2nd pinnules have square shape (Figs. 4A, 4B), while the 4th, 5th, 6th and 7th pinnules have trapezoid shape (Figs. 4C–4F), this is because the 4th, 5th, 6th and 7th pinnules have more spaced basal processes in comparison with the 1st and 2nd pinnules; the 1st, 2nd, 4th and 5th pinnules display winged lateral processes on both sides (Figs. 4A–4D), but lateral processes are not present in the 6th and 7th pinnules (Figs. 4E, 4F); although all pinnules present posterior processes on both sides the 4th and 5th pinnules present more protuberant ones in comparison with other pinnules (Figs. 4C, 4D).

### C. calabaricus

**Figure 5:**
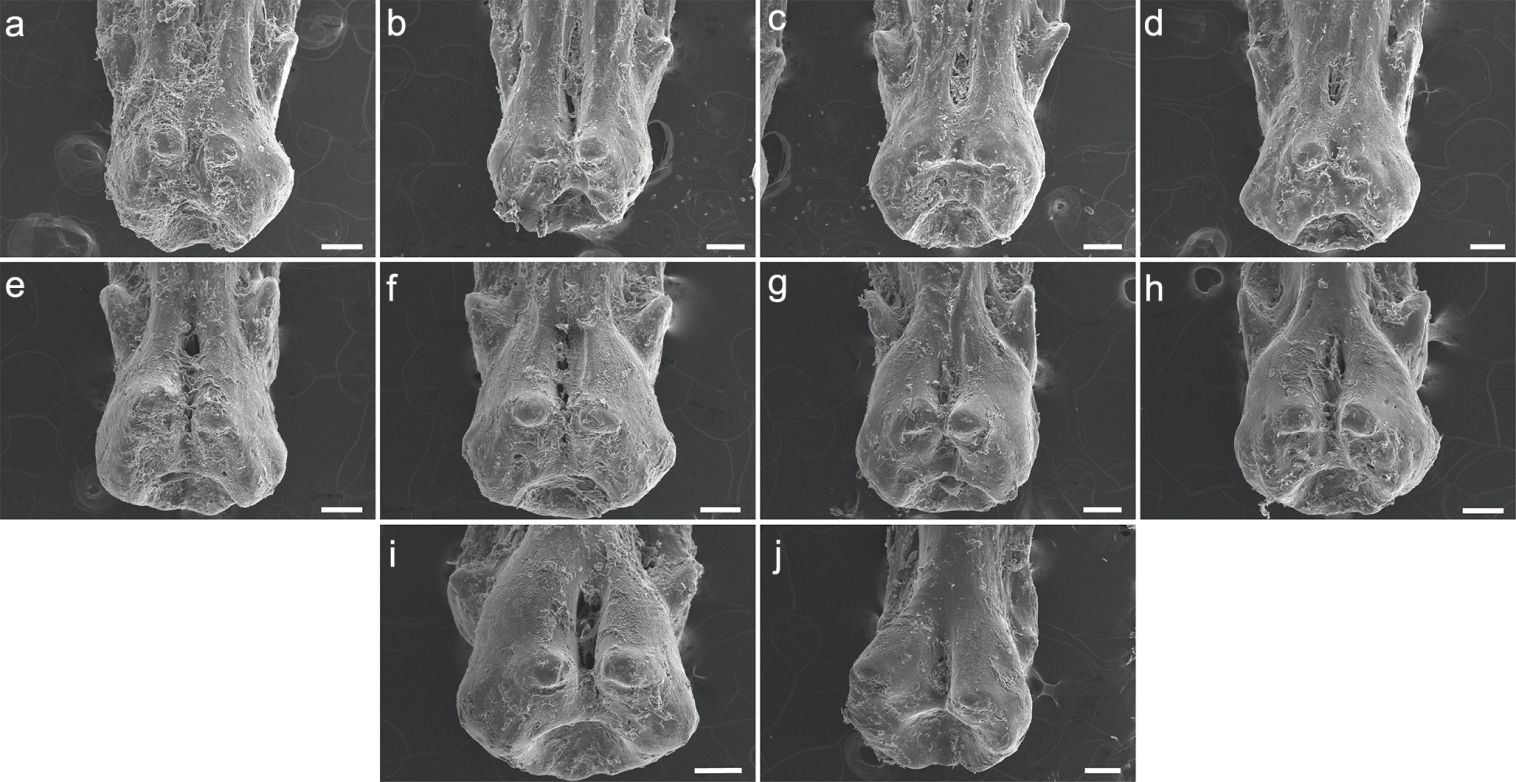
SEM images of the pinnules of *C. calabaricus* UERJ-PNT 527. (A–J) corresponds to the 1st, 2nd, 3rd, 4th, 5th, 6th, 7th, 8th, 9th and 10th pinnules, respectively. Scale = 200 µm).

**Figure 6:**
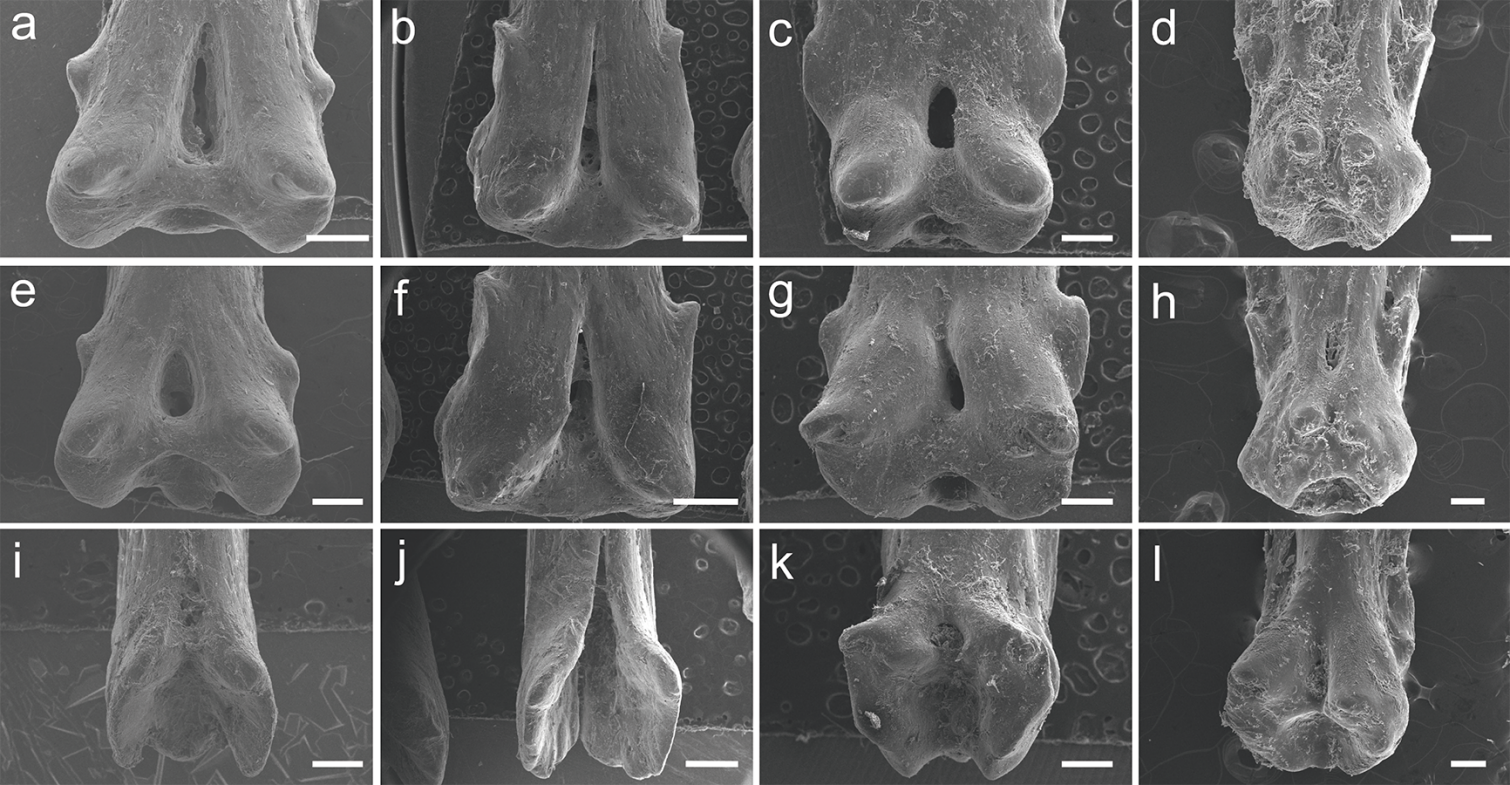
SEM images of the pinnules of four extant polypterids. SEM images of the pinnules of *P. delhezi* PNT 525 (A, B and C) 1st, 6th and 11th pinnules, respectively. Scale = 500 µm), *P. endlicherii* PNT 522 (D, E and F) 1st, 6th and 12th pinnules, respectively. Scale = 1 mm), *P. palmas* PNT 526 (G, H and I) 1st, 4th and 7th pinnules, respectively. Scale = 500 µm) and *C. calabaricus* PNT 527 (J, K and L) 2nd, 6th and 10th pinnules, respectively. Scale = 200 µm).

The specimen UERJ-PNT 527 had 10 pinnules. All pinnules share great similarity, mainly due to their rounded shape, their rounded basal processes and the tiny posterior process placed very close to the foramen (Fig. 5). The differences among them are the lateral process (all pinnules present lateral processes on both sides), where the 10th pinnule presents a rounded lateral process edge (Fig. 5J) and all the other present pointed lateral processes edge (Figs. 5A–5I). The posterior processes are potentially absent at least on one side in the 2nd, 3rd, 4th, 5th, 10th pinnules (Figs. 5B–5E, 5J); and the basal foramen is elongate in the 2nd, 3rd, 5th, 6th and 9th pinnules (Figs. 5B, 4C, 4E, 4I) and rounded in the 4th, 7th and 10th pinnules (Figs. 5D, 5G, 5J). The basal foramen of the 1st and 8th pinnules could not be observed (Figs. 5A, 5H). Additionally, the pinnules of *C. calabaricus* differ substantially from all the pinnules of the three *Polypterus* species for their overall rounded shape and more prominent lateral processes (Fig. 6).

## Discussion

Morphological variations comprise interspecific and intraspecific variations and are mainly divided into taxonomic, ontogenetic, and individual types (Grande, 2004). The observation of these variations is crucial for taxonomic and phylogenetic studies. However, throughout the fossil record, the size and quality of the sample is critical to remark these variations within different taxa.

Several taxa of fossil polypteriforms are mainly described based on disarticulated material, usually on the articular head of pinnules (Gayet & Meunier, 1996; Gayet, Meunier & Werner, 1997; Werner & Gayet, 1997; Daget et al., 2001). To date, six genera, including 17 species, were described based only on the articular head of the pinnules, most of them from the same formations and localities (Gayet & Meunier, 1996; Werner & Gayet, 1997). Despite the great number of described taxa based on pinnule morphology, there is no study that proves the viability of this character as genera or species level diagnosis.

Here we tested in various species of the extant *Polypterus* and *C. calabaricus* the morphology of the pinnules and describe for the first time non taxonomical variations among them. We put in question the identification and validation of some polypterifoms fossil taxa that were described based exclusively on the different morphology of the pinnules, and do not take in consideration the potential individual variation, nor the possible deformation caused by the fossilization process. The material described here displays interspecific and intraindividual variations; in particular the pinnules have different morphology according to the position in the dorsal fin (Fig. 6). Nevertheless, when comparing two different specimens of *P. delhezi* there is little or no interindividual variation, in particular the morphology of pinnules remains conservative according to its position (see Fig. S1), suggesting that this character may be also relevant as a taxonomic feature for diagnosis.

In all the species examined here, pinnules are somewhat symmetrical (*contra* few fossil taxa known in the Cretaceous of Africa based on asymmetrical pinnules) and the variation observed in the pinnules of each specimen (Fig. 6) is quite substantial and points to an intraindividual variation. The condition may have been interpreted as taxonomic variation in isolated fossil material (c.f. Grande, 2004), resulting in bogus taxonomic interpretations for isolated pinnules, parataxonomy and, consequently, overestimation of the number of taxa (Krell, 2004). As a result it is very clear that multiple fossil species might be erected for different pinnules from the same taxon. A revision of these fossil taxa is needed, as well as a complete study of the living species pinnules.

## Conclusions

Despite being used in paleontological studies of taxonomy, the presence of morphological variations among the pinnules of extant polypterids partially questions the validity of these structures as morphological characters.

The pinnules of the species described here display interspecific differences and intraindividual variations, depending on the position along the dorsal fin. On the other hand, there is very few or no interindividual variation, which may suggest that some of these characters could be relevant for diagnosis. However, this statement should be confirmed by a larger sample.

As most of the fossil polypterids are described based on the articular head of the pinnules, we strongly suggest caution when describing new taxa, especially when different fragments occur in the same locality.

##  Supplemental Information

10.7717/peerj.5083/supp-1 Figure S1 SEM images of the pinnules of *P. delhezi* UERJ-PNT 528 A–K corresponds to the first, second, third, fourth, fifth, sixth, seventh, eighth, ninth, tenth and eleventh pinnules, respectively. Scale = 500 µm.

## Additional Information and Declarations

### Data Availability

The following information was supplied regarding data availability: All the specimens are permanently stored in the collection of the Universidade do Estado do Rio de Janeiro.
