# Supplementary figures and images for: ­Morphological variations in the dorsal fin finlets of extant polypterids raise questions about their taxonomical validity
Source: PeerJ. 2018 Jun 29;6:e5083. doi: 10.7717/peerj.5083 (PMC6056263; doi:10.7717/peerj.5083)

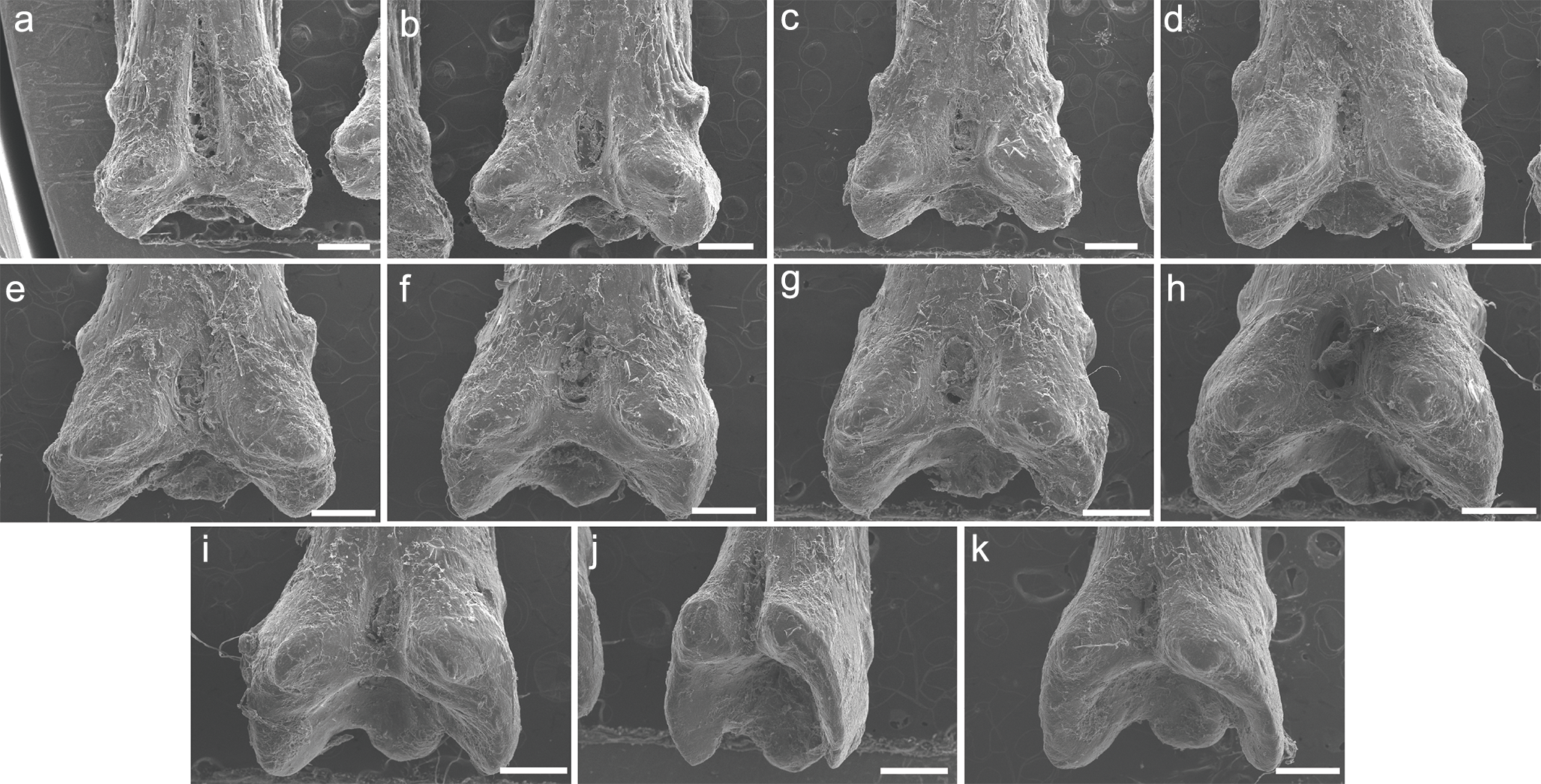

Supplement: Figure S1 — A–K corresponds to the first, second, third, fourth, fifth, sixth, seventh, eighth, ninth, tenth and eleventh pinnules, respectively. Scale = 500 µm. [file peerj-06-5083-s001.png]
